# Penetrance of HNPCC-related cancers in a retrolective cohort of 12 large Newfoundland families carrying a MSH2 founder mutation: an evaluation using modified segregation models

**DOI:** 10.1186/1897-4287-7-16

**Published:** 2009-10-28

**Authors:** Karen A Kopciuk, Yun-Hee Choi, Elena Parkhomenko, Patrick Parfrey, John McLaughlin, Jane Green, Laurent Briollais

**Affiliations:** 1 Division of Population Health Research, Alberta Health Services Board, Calgary, Alberta, Canada; 2 Department of Epidemiology and Biostatistics, The University of Western Ontario, London, Ontario, Canada; 3 Research Institute, Hospital for Sick Children, Toronto, Ontario, Canada; 4 Clinical Epidemiology Unit, Memorial University, St John's, Newfoundland, Canada; 5 Population Studies and Surveillance, Cancer Care Ontario, Toronto, Ontario, Canada; 6 Samuel Lunenfeld Research Institute, Mount Sinai Hospital, Toronto, Ontario, Canada; 7 Discipline of Genetics, Memorial University, St John's, Newfoundland, Canada

## Abstract

**Background:**

Accurate risk (penetrance) estimates for associated phenotypes in carriers of a major disease gene are important for genetic counselling of at-risk individuals. Population-specific estimates of penetrance are often needed as well. Families ascertained from high-risk disease clinics provide substantial data to estimate penetrance of a disease gene, but these estimates must be adjusted for possible specific sources of bias.

**Methods:**

A cohort of 12 independently ascertained HNPCC families harbouring a founder MSH2 mutation was identified from a cancer genetics clinic in St. John's, Newfoundland, Canada. Carrier status was known for 247 family members but phenotype information on up to 85 additional relatives with unknown carrier status was available; using modified segregation models these additional individuals could be included in the analyses. Three HNPCC-related phenotypes were evaluated as age at diagnosis of: any HNPCC cancer (first cancer), colorectal cancer (CRC), and endometrial cancer (EC) for females.

**Results:**

Lifetime (age 70) risk estimates for male and female carriers were similar for developing any HNPCC cancer (Males = 98.2%, 95% Confidence Interval (CI) = (93.8%, 99.9%); Females = 92.8%, 95% CI = (82.4%, 99.1%)) but female carriers experienced substantially reduced lifetime risk for developing CRC compared to male carriers (Females = 38.9%, 95% CI = (24.2%, 62.1%); Males = 84.5%, 95% CI = (67.3%, 91.3%)). Female non-carriers had very low lifetime risk for these two outcomes while male non-carriers had lifetime risks intermediate to the female carriers and non-carriers. Female carriers had a lifetime risk of developing EC of 82.4%. Relative risks for developing any HNPCC cancer (carriers relative to non-carriers) were substantially greater for females compared to their male counterparts (Females = 54.8, 95%CI = (4.4, 379.8); Males = 9.7, 95% CI = (0.3, 23.8)). Relative risks for developing CRC at age 70 were substantially greater for females compared to their male counterparts (Females = 23.7, 95%CI = (5.6, 137.9); Males = 6.8%, 95% CI = (2.3, 66.2)). However, the risk of developing CRC decreased with age among both genders.

**Conclusion:**

The proposed modified segregation-based models used to estimate age-specific risks for HNPCC phenotypes can reduce bias due to ascertainment and missing genotype information as well as provide estimates of absolute and relative risks.

## Background

Extensive knowledge about Mendelian disease genes is accumulating as more are discovered, characterized and studied in high risk families. With this accumulating knowledge comes the opportunity for carriers to have improved surveillance and treatment options so disease is detected at an early stage and adverse outcomes are reduced. However, accurate age-and sex-specific penetrance estimates are critical for genetic counseling of at-risk individuals in order to establish and evaluate disease control efforts. For instance, risk models have recently been developed to predict germline mutations linked to Lynch syndrome (hereditary nonpolyposis colorectal cancer or HNPCC) but unlike existing models, these models require a prior specification of the penetrance function to give accurate predictions [1-3].

HNPCC is an example of an autosomal dominant Mendelian disease which has seen remarkable improvements in associated morbidity and mortality in recent years due to early identification, surveillance and treatment of gene carriers [4,5]. It is caused by a mutation in one of the mismatch repair genes: MLH1, MSH2, MSH6 or PMS2 and accounts for about 3% of all colorectal cancer (CRC) cases [6]. Carriers of one of these mutations are at increased lifetime risk of developing cancers of the colon, endometrium, ovary, stomach, ureter, upper biliary tract, skin, and brain [7,8]. Early age at onset is another hallmark of this syndrome.

Studies of heritable genetic diseases are often conducted using families with multiple affected individuals since high risk alleles are often rare in the general population. However, families identified through high risk disease clinics tend to be more likely to carry the disease gene mutation and to have phenotype information, compared to pedigrees identified through population-based sampling of affected probands. Thus, families identified in clinic-based designs may not be representative of the population and ascertainment may be biased to multiple case families. In addition to ascertainment issues, missing genotype information can be problematic in these pedigrees. Genetic testing may not be offered to all family members based on an individual's risk of carrying the deleterious allele and some family members may have already died without testing. Still others may decline testing.

Statistical models based on segregation methods have been developed which can estimate age-specific penetrance in these high risk families and can adjust for ascertainment bias and missing genotype data. Traditionally, segregation-based methods have been used to fit genetic models to phenotype data collected from pedigrees. These methods can easily adjust for complex ascertainment when the rules of sequential sampling of pedigrees are followed, infer the missing genotypes using family structure and evaluate whether another segregating gene might be involved [9,10]. More precise estimates and confidence intervals (CIs) can be obtained with these methods compared to standard methods, since additional genotyped individuals are included. When the form of the disease risk (e.g., risk increasing or decreasing with age or both) could vary for different age-at-onset phenotypes, a general formulation of the hazard function is necessary.

This paper addresses these key challenges in disease risk estimation by using new statistical methods in a study of 12 HNPCC families, who were ascertained at a Newfoundland (NL) cancer genetics clinic and who share a founder MSH2 mutation. In Newfoundland, founder mutations causing autosomal dominant disease, large family sizes over 8-10 generations and little in or out migration since the founding settlements in the late 18^*th *^and early 19^*th *^centuries [11] have enabled discoveries of phenotype - genotype correlations in right arrhythmogenic ventricular cardiomyopathy, [12] autosomal dominant polycystic kidney disease, [13] and HNPCC [14]. These NL families share common lifestyle and/or environmental factors which makes them well-suited for genetic studies of risk estimation. However, their isolation and development might mean that risk estimates from other populations may not be appropriate for them as other risk factors in addition to the founder mutation may affect the rates of penetrance [15-18].

## Methods

### Family ascertainment and characteristics

Twelve families with hereditary CRC were independently identified from the Medical Genetics Clinic at Memorial University, St. John's, NL, Canada, and were confirmed as carrying the MSH2 mutation A → T nt942+3. Based on their geographic proximity in an isolated area of the province, as well as having common haplotypes of a subset of at least five microsatellite markers, it is assumed these families have a common ancestor. See Green et al. (2002) for additional details [7]. Data collected from these families have been updated since that initial report, including additional HNPCC outcomes and genotyping of family members.

DNA testing for the MSH2 mutation identified individuals as carriers or non-carriers. For those not tested, clinically presumed (including obligate) carriers for any HNPCC were evaluated using Bayes-Mendel [19]. Using a carrier probability threshold of 0.95, 36 presumed carriers were identified as carriers. All other individuals were considered to have unknown mutation status.

### Statistical Methods

The clinical outcomes of interest (phenotypes) in this study were age to diagnosis of CRC, age to diagnosis of first HNPCC cancer (included CRC, small bowel, endometrium, ovary, gastric, urinary tract, brain, and bile duct), and among the females, age to diagnosis of endometrial cancer (EC). Lifetime risk or penetrance at age 70 for these three outcomes is of particular interest. Observations on participants were censored at their age at last followup if no phenotypes were observed; after entering a screening program; or for age to diagnosis of EC for females, after having a hysterectomy/oophorectomy for non-cancer reasons.

The Kaplan-Meier (KM) estimator is often used to estimate age-specific penetrance but has known limitations [20]. Although it is robust to the correlation in outcomes among family members, it does not correct for ascertainment bias associated with these high risk families or infer missing genotype information.

Modified segregation-based methods can account for the non-random ascertainment of families as well as missing genotype information [9,10,21]. We considered a general parametric form for the hazard function, which adopted a Weibull baseline hazard function. The generalized log-Burr hazard form (see Additional file 1) includes the standard Weibull proportional hazards model or the log-logistic model as special cases [22]. The Weibull model is quite flexible but does have a monotonic functional form of the hazard whereas the log-logistic specification does not. As Jenkins et al. (2006) found, risk can increase and then decrease with age, so assuming monotonicity for risk may not always be appropriate [23]. With three clinical phenotypes of interest, the general formulation permits appropriate functional form choices for each outcome. Likelihood ratio statistics were used to select a simpler model (i.e., either Weibull or log-logistic) that was compatible with the general model [24]. All regression models included the variables for sex and genotype status and their interaction.

Stratified risk estimates were calculated by sex and by mutation status for each phenotype and the corresponding 95% confidence intervals were calculated by simulation. Specifically, 1,000 parameters sets were simulated, assuming a multivariate normal distribution for the estimated parameters. The 95% CI for the cumulative risk to a specific age (range 20-70 years) was estimated as the region between the 2.5^*th *^and 97.5^*th *^percentiles of the distribution obtained by substituting the sets of simulated parameters into the penetrance function [23].

To correct penetrance estimates for families ascertained through an individual with cancer who also satisfied high or intermediate familial risks, we used an ascertainment-corrected retrospective (ACR) likelihood approach (see Additional file 2) [25-27]. Correction was based on the conditional probability of the observed genotypes in the first degree relatives of the carrier proband, given the observed presence or absence of disease in each person, and corrected for the ascertainment event (i.e. the diagnosis of cancer in the proband) which caused the family to be sampled [9,10,25]. We obtained the maximum likelihood estimates of the needed parameters by maximizing this ACR likelihood and then used the parameters to estimate age-specific cumulative and relative risks for each outcome, using the penetrance function in equation (2) of Additional file 1[9,10]. These modified segregation methods based on an ACR likelihood were implemented in the genetic software program, Mendel [28].

## Results and Discussion

### Description of Family Data Set

The number of family members with phenotype information varied from 146 (EC) to 313 (any HN-PCC cancer) to 332 (CRC), with 122 males and 125 females having known genotypes (see Table 1). However, family members with unknown mutation status often had a substantial number of outcomes. For instance, 15 of the 43 males with unknown carrier status were diagnosed with having any HNPCC cancer. Excluding these family members who were not genotyped and were not presumed carriers will impact the risk estimates.

**Table 1:** Description of NL Family Data Set.

**Group**	**Any HNPCC^† ^cancer**		**CRC^‡^**		**EC^Δ^**	
	**Events**	**n**	**Events**	**n**	**Events**	**n**
	
**Males**						
Carriers*	34	71	32	71	-	-
Non-carriers	2	51	1	51	-	-
Unknowns	15	43	9	53	-	-
**Females**						
Carriers*	38	74	16	74	16	73
Non-carriers	1	51	0	51	0	51
Unknowns	9	23	4	32	1	22
	
Family Members with Phenotype Information		313		332		146

Number of each phenotype per subgroup based on sex and mutation status

* includes known carriers and presumed carriers, n = number of relatives

^† ^HNPCC = hereditary nonpolyposis colorectal cancer

^‡ ^CRC = colorectal cancer, ^Δ ^EC = endometrial cancer

### Modified Segregation-based Methods

The general formulation for the hazard function (shown in Additional file 1 and which included the interaction term) yielded these findings:

• Any HNPCC cancer: log-likelihood value was -617.3 and  was 13831,

• CRC: log-likelihood value was -479.1 and  was 0.96,

• EC: log-likelihood value was -283.4 and  was 0.2.

These results suggest that different models are appropriate for each phenotype. Likelihood ratio test statistics confirmed that the general model formulation was consistent with these specific parametric forms: the Weibull model for any HNPCC cancer (p-value = 1.0, log-logistic model had p-value of 0.03), and the log-logistic model for CRC (p-value = 0.40, Weibull model had p-value of 0.11, but had a larger log likelihood value). The log-logistic model was used for EC (p-value = 0.03), as the general model did not converge.

**Figure 1:**
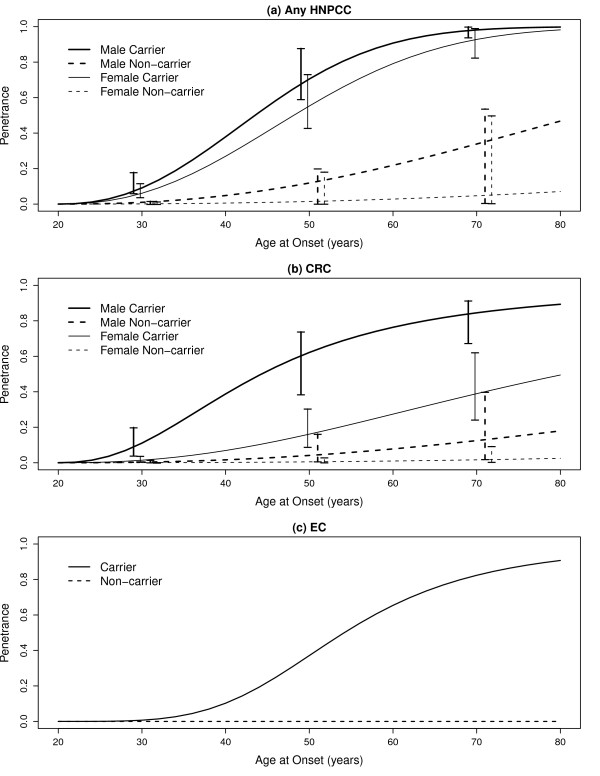
**Segregation model estimates of penetrance for any HNPCC, CRC and EC**. Age-specific cumulative risks and 95% confidence intervals of hereditary nonpolyposis colorectal cancer related cancers [(a) any hereditary nonpolyposis colorectal cancer, (b) colorectal cancer, (c) endometrial cancer] among mutation carriers and non-carriers specified by gender, based on the segregation analyses.

Based on these selected models for each phenotype, we estimated the penetrance functions using the corresponding parameter estimates. The estimated penetrance functions, stratified by sex and mutation status, are displayed in Figure 1. The age-related risk of developing any HNPCC cancer for carriers increases quickly after age 30, with females experiencing slightly lower risks than males over their lifetimes. Male non-carriers also experience slightly greater lifetime risk than the female non-carriers with differences appearing past age 40.

Cumulative risk for CRC was not surprisingly highest for males, compared to their female counterparts. For instance, lifetime CRC risk estimates for male carriers was 84.5% (95% CI = (67.3%, 91.3%)) whereas for female carriers it was 38.9% (95% CI = (24.2%, 62.1%)). The 95% CIs drawn at ages 30, 50 and 70 reveal that male carriers have significantly higher plausible values than female carriers, but there is no difference between genders among the non-carriers. Table 2 provides the point and 95% CI estimates of the penetrance for any HNPCC cancer, CRC and EC at ages 30, 50 and 70 (lifetime risk).

**Table 2:** Penetrance and 95% Confidence Interval Estimates.

**Group**	**Age 30 (95% CI)**	**Age 50 (95% CI)**	**Age 70 (95% CI)**
**Any HNPCC cancer (Weibull)**			
Male Carrier	9.1 (6.0, 17.9)	70.5 (59.1, 87.7)	98.2 (93.8, 99.9)
Female Carrier	6.1 (3.8, 11.6)	55.3 (42.8, 73.1)	92.8 (82.4, 99.1)
Male Non-carrier	1.0 (0.0, 1.6)	11.9 (0.2, 20.0)	33.9 (0.5, 53.6)
Female Non-carrier	0.1 (0.0, 1.4)	1.5 (0.1, 18.2)	4.7 (0.4, 49.8)

**CRC (Log-logistic)**			
Male Carrier	11.1 (3.9, 19.9)	62.2 (38.4, 73.8)	84.5 (67.3, 91.3)
Female Carrier	1.4 (0.6, 3.7)	16.1 (8.9, 30.5)	38.9 (24.2, 62.1)
Male Non-carrier	0.3 (0.0, 1.6)	4.1 (0.6, 16.2)	12.5 (2.0, 39.8)
Female Non-carrier	0.0 (0.0, 0.3)	0.5 (0.1, 2.9)	1.6 (0.3, 9.3)

**EC (Log-logistic)**			
Female Carriers	0.7	37.1	82.4
Female Non-carriers	0.0	0.0	0.0

Penetrance estimates (percent) at ages 30, 50 and 70 by carrier status and sex, with corresponding 95% confidence intervals. Estimates based on the specified hazard function for age at diagnosis of any hereditary nonpolyposis colorectal cancer (any HNPCC cancer), colorectal cancer (CRC) and endometrial cancer (EC)

Endometrial cancer results reveal a relatively low cumulative risk to age 30 (0.7%) for female carriers which rises sharply to 82.4% by age 70. For non-carriers, risk was essentially zero throughout the women's lifetimes. The low number of women diagnosed with EC meant confidence intervals could not be estimated.

In addition to the absolute risk estimates obtained with the modified segregation analyses, the relative risks (RR) for developing any HNPCC cancer and CRC were also calculated (Equation 2, Additional file 1). Table 3 presents the risks for carriers relative to non-carriers, stratified by sex. For age at onset of any HNPCC cancer, the Weibull model was again adopted so the RR are automatically constant with respect to age. The interaction between carrier status and sex was not significant in this model. Although males had higher absolute risks compared to females regardless of carrier class, female carriers had more than five times the relative risks (54.8), compared to male carriers (9.7). The very wide 95% CI for the female carriers (4.4, 379.8), however, suggests a lack of precision; it also overlaps with the corresponding ones for the male carriers (0.3, 23.8) indicating no statistically significant difference between them. The scant number of events in female non-carriers meant RR estimates for EC were not estimable.

**Table 3:** Hazard Ratio and 95% Confidence Interval Estimates.

	**Group**	**Age 30 (95% CI)**	**Age 50 (95% CI)**	**Age 70 (95% CI)**
**Any HNPCC cancer **(Weibull)				
	Male	9.7 (0.3, 23.8)	9.7 (0.3, 23.8)	9.7 (0.3, 23.8)
	Female	54.8 (4.4, 379.8)	54.8 (4.4, 379.8)	54.8 (4.4, 379.8)

**CRC **(Log logistic)				
	Male	34.1 (7.1, 167.0)	15.1 (3.9, 110.2)	6.8 (2.3, 66.2)
	Female	37.7 (7.5, 176.9)	32.2 (6.9, 162.1)	23.7 (5.6, 137.9)

Hazard ratio estimates at ages 30, 50 and 70 in mutation carriers compared with mutation non-carriers by sex, with corresponding 95% confidence intervals. Estimates based on the specified hazard function for age at diagnosis of any hereditary nonpolyposis colorectal cancer and colorectal cancer

HNPCC = hereditary nonpolyposis colorectal cancer

CRC = colorectal cancer

For age at onset of CRC, the log-logistic model was adopted so risks could vary with age. Overall, the risk for female carriers relative to female non-carriers was higher than for male carriers relative to male non-carriers over the entire age range considered. At age 30, the RR for male carriers, 34.1 (95% CI = (7.1, 167.0)) was nearly the same as for female carriers, 37.7 (95% CI = (7.5, 176.9)). But by age 70, the relative risk for male carriers (6.8, 95% CI = (2.3, 66.2)) was one third the relative risk for female carriers (23.7, 95% CI = (5.6, 137.9)). Thus, female carriers had greater relative risks of developing CRC compared to their male counterparts through out their adult lifetimes although their 95% CIs did overlap. This risk difference became substantial by age 70.

## Discussion

Our paper confirmed the relatively high penetrance associated with MSH2 gene mutations in these NL HNPCC families for various phenotypes, including CRC. Several studies have previously evaluated the risk of developing CRC among identified MMR gene mutation carriers in HNPCC families, where the risk to age 70 ranged from 22% to 100% [7,15-18,29-36]. Some studies suggested different risks associated with MLH1 and MSH2 [16,31] while others reported males having nearly twice the risk of females [15,16,18]; these differences were not consistent across studies. The lower penetrance reported among females suggests they are somehow protected from CRC, perhaps due to environmental/reproductive factors unique to women or to a sex-linked modifier gene. However, not all of these studies adjusted for ascertainment, nor adopted the same statistical methods of penetrance function estimation.

Our study confirmed a gender-specific effect on the risk of developing CRC with a higher lifetime risk in males than females (84.5% vs. 38.9% by age 70). These high risks could be due to the specific founder mutation effect, shared environment and limited screening for many of the older family members. Our study also underlined the relative importance of other cancers in these large Newfoundland families. Women are susceptible to developing endometrial cancer with a penetrance close to 82.4% by age 70 and when considering all HNPCC-related cancers, the lifetime risks in males and females carriers were similarly very high, 98.2% and 92.8%, respectively. Interestingly, our study also showed that the relative effect of MSH2 on CRC for males and females combined as measured by the hazard ratio, decreases with age (from 43.1 at age 30 to 16.9 at age 70, results not shown). Such an effect was also suggested in Jenkins et al. [23] and in a very recent study from Ontario [9].

To illustrate the importance of having specific penetrance estimates for the HNPCC families in Newfoundland (NL), we estimated both the probability of being a MSH2 mutation carrier and the probability of developing CRC and EC in cancer-free individuals in Family 1 from our sample. These probabilities can be computed for any family member, i.e. the counselee, and are conditional on the observed phenotypes and genotypes in the family. Gender- and age-specific penetrance are used to derive a posterior probability of being a mutation carrier and then the disease risk estimate is calculated as a weighted average over the mutation status, where the weights are the mutation carrier probabilities [19]. Computations were carried out using the package MMRpro in the R library BayesMendel [19]. We compared the probability of being a mutation carrier and of developing the two outcomes for a selected counselee using either publically available penetrance estimates for MSH2 [1] or the penetrance estimates obtained from our own analyses of the NL family data set.

**Figure 2:**
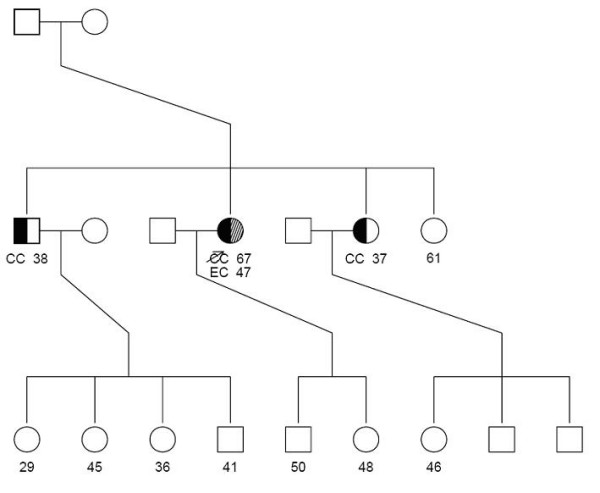
**Family 1**. Pedigree illustrating need for population-specific probabilities of being a MSH2 mutation carrier and the probability of developing CRC and EC in cancer-free individuals in Family 1 from our sample.

We chose individual 48 in family 1 as the counselee (see Figure 2). This is a woman who is cancer free to age 48 and is a mutation carrier. Her mother (the proband in the family) had an endometrial cancer (EC) at age 47 and a colorectal cancer (CRC) at age 67 and was a confirmed mutation carrier. First, we ignore the counselee's mutation status. Assuming a mutation prevalence varying from 0.1% to 1% in the Newfoundland population, the carrier probability for the counselee ranged from 37% to 41% when using our derived penetrance estimates and was similar to the value of 43% obtained from using the published estimates. The probability of developing CRC and EC by age 83 for this person was 18% and 28%, respectively, when using our penetrance estimates and 12% and 19%, respectively, when using the published estimates. Next, we assumed the couselee's mutation status is known. The probability of developing CRC and EC by age 83 was 42% and 70%, respectively, when using our penetrance estimates but only 22% and 40%, respectively, when using the published penetrance estimates. These risk estimates were not sensitive to the mutation prevalence. Thus, using our calculated penetrance values, we found both the carrier probability and development of both phenotypes differed from values obtained using published data from other populations.

Our paper also demonstrates the strength of the modified segregation-based analysis to estimate the penetrance of HNPCC-related cancers in these large Newfoundland families. First, unlike the classical Kaplan-Meier estimator, this approach can infer missing genotypes by using the Mendelian transmission probabilities and genealogical relationships. As a consequence, the segregation-based analysis was able to use information on up to 85 additional relatives, resulting in more precise and potentially less biased penetrance estimates. Second, the modified segregation-based analysis can correct for ascertainment when families were recruited through several affected individuals. Our recent simulation studies [9] have shown that the inference through the retrospective ascertainment-corrected likelihood approach that we proposed was nearly unbiased for various types of family-based designs (population-and clinic-based) and genetic models (including one-and two-gene models). Finally, segregation-based methods allow several parametric hazard functions to be considered. We proposed a generalized log-Burr formulation of this function, which includes the Weibull or log-logistic forms as particular cases, so a better fit of the cumulative risk function results.

For the aforementioned reasons, our results differ somewhat from those published by Green et al. [7] who used the Kaplan-Meier estimator on an earlier version of the data. For example, their penetrance estimates for CRC were 92% and 64% by age 70 in males and female carriers, respectively, compared to 85% and 39% in our study. The main difference is likely from our use of an ascertainment correction [25]. In addition, Green et al. (2002) combined the relatives with unknown genotypes with the non-carriers, whereas our approach used a weighted average over the missing data.

Our results also differ from those of Quehenberger et al. (2005), Barrow et al. (2008) and Alarcon et al. (2007), even though these three studies also adjusted for ascertainment of high-risk families and missing genotype information [33,35,36]. Several important differences might explain the lower risk estimates these authors found in their data. Although all of these other studies had substantially more confirmed carrier families for the phenotypes they considered (CRC, and possibly EC and Minor HNPCC site), they ended up combining families segregating different DNA mismatch repair (MMR) alleles together. They also had proportionally more missing genotype information in the MSH2 families than the 34% in our data: 68.3% in Quehenberger et al. (2005), 41 to 76% in Barrow et al. (depending on whether one includes the putative and assumed carriers as known) and 84% in Alarcon et al. (in the combined MLH1 and MSH2 families). All of the families in these other studies of non-island populations likely did not share the same founder mutation, as was the case in the NL data.

In addition to the differing study populations, pooling across MMR genes and varying proportions of genotyped family members, methodological differences might also be impacting the penetrance estimates. Quehenberger et al. and Alarcon et al. assumed a fixed population allele frequency and adopted their national (Netherlands or France) cancer incidence rates for the noncarriers rather than estimating it. Modelling differences between these studies and this one included the use of polynomial functions assuming competing risks (Quehenberger et al.), a genotype restricted likelihood method that employed a Weibull distribution (Alarcon et al.), and the Kaplan-Meier nonparametric estimator (Barrow et al.).

The competing risk model estimates, in particular, are affected by the rates of all phenotypes under consideration, which are also likely not independent for HNPCC cancers. Absolute risk estimates for the time to first HNPCC cancer from our study (Table 2, first two rows) can be compared to Quehenberger et al. combined (CRC + EC +MC) age dependent cause specific cumulative risks (Table four, second section, last two columns): our risk estimates at ages 30, 50 and 70 years are consistently higher for both male and female carriers although both approaches had wide 95% confidence intervals. Relative risks for time to first CRC (Table 3 in both studies) suggest decreasing risk over lifetimes, with our study estimating a wider range of values and different values for males and females. Our absolute lifetime risk estimates for time to CRC from our study can be compared to those of Barrow et al. (Table four, rows 2 and 4, column 70-79 years): our risk estimates at age 70 years are higher for male MSH2 carriers but lower for female carriers and our 95% confidence intervals substantially wider. When we compare our estimates of time to CRC with the results obtained by Alarcon et al. (Figure 2, combined MSH2 and MLH1 families), our risk estimates at ages 30, 50 and 70 years are consistently higher for both male and female carriers but the wide 95% confidence intervals do overlap for each gender. Estimates of time to EC are also much higher in our study than in the Alarcon et al. one, although both found very little risk until age 30.

## Conclusion

In summary, the risk estimates we obtained using a modified segregation approach within a general hazard framework are adjusted for ascertainment of the family members and are able to include those family members with missing genotype information. Thus, this novel and flexible approach reduces several sources of bias in the penetrance estimates for HNPCC-related phenotypes. However, the most appropriate ascertainment adjustment and method for dealing with missing genotype information for penetrance estimation is an open problem.

Although many sources of bias have been reduced in this study, several limitations may still exist. First, the sample size is relatively small for a risk estimation study and the confidence intervals were often wide, especially for estimating gender-specific penetrances. This problem was only partly overcome by using the modified segregation-based approach. Second, inaccuracy of cancer diagnosis or screening history might introduce error because some cases or dates of entry into screening programs could not be confirmed. Further work on the impact of screening is warranted. Lastly, we did not adjust our analysis for competing risks nor try to model specifically the correlation between the different HNPCC-related cancers or other sources of familial correlation besides the MSH2 mutation. Phenotypic heterogeneity and multiple phenotypes also pose challenges. We are planning to investigate these issues in some future work.

## Competing interests

The authors declare that they have no competing interests.

## Authors' contributions

LB conceived of the study. KAK and Y-HC developed the statistical methods and then performed the statistical analyses. The manuscript was drafted by KAK. LB provided oversight of the data analyses and critical revision of the manuscript for its intellectual content. JG and PP designed the original study and continue to coordinate follow-up data collection. All authors read and approved the final manuscript.

## Supplementary Material

Additional file 1 **General hazard specification for parametric regression model**. The formulation of the hazard function based on a generalized log-Burr specification of the survival function. Click here for file

Additional file 2 **Retrospective Ascertainment-Corrected Likelihood**. Details on the derivation of the correction for ascertainment of high risk families. Click here for file
